# Health of the corporate worker: health risk assessment among staff of a corporate organization in Ghana

**DOI:** 10.1186/s12995-015-0072-7

**Published:** 2015-08-12

**Authors:** Henry Aidoo, Akye Essuman, Phyllis Aidoo, Anita O. Yawson, Alfred E. Yawson

**Affiliations:** 125 Row House, Michener Park, Edmonton, AB T6H, 4M4 Canada; Family Medicine Unit, Department of Community Health, School of Public Health, College of Health Sciences, University of Ghana, Korle-Bu, Accra Ghana; Department of Anaesthesia, Korle-Bu Teaching Hospital, Korle-Bu, Accra Ghana; Department of Community Health, School of Public Health, College of Health Sciences, University of Ghana, Room 46, P. O. Box 4236, Korle-Bu, Accra Ghana

**Keywords:** Health promotion, Corporate worker, Risk assessment, Ghana, Low income setting

## Abstract

**Introduction:**

Health promotion at the workplace and for workers is important to promote workers’ health, improve working environments and work practices. The goal of this analysis was to provide an example of health risk assessment conducted in a large media organization in Ghana for its workers and to identify correlates of health risks identified among different categories of workers.

**Methods:**

This was a cross sectional study of the health risk of staff in a large corporate media organization in Accra, Ghana, conducted in 2012. In all 161 members of staff were screened and records included in the analysis. An abstraction form was used to collect data on age and sex of staff, staff category, self-reported health risk, history of chronic disease and self-rated health status. Measurements included weight, height, Body Mass Index, fasting blood sugar, total cholesterol/ HDL cholesterol and blood pressure. Data were entered into SPSS version 21, and analyzed by simple frequencies, proportions and ratios. Measured health indices were analyzed by mean ± standard deviation. Significant association between categorical outcome measures were determined with chi-square test at the 95 % confidence level.

**Results:**

The sex characteristics of the workers indicated more males than females, male: female sex ratio of 2.3: 1. Close to half of the workers 66 (41.0 %) self-reported history of chronic disease and 40 (24.8 %) self-rated their overall state of health as poor. In all, 31.7 % of workers self-reported hypertension, while measured blood pressure indicated 60.2 % prevalence of diastolic blood pressure. Prevalence of obesity was 63.8 %; 49.1 % of staff had above normal total cholesterol levels and 12.4 % had blood glucose indicative of diabetes. Senior and management staff had relatively higher prevalence of obesity, high blood pressure, above normal cholesterol and fasting blood sugar levels.

**Conclusion:**

More staff were unaware of their individual health risks and the health risks were higher among senior staff and management members. Adoption of regular health educational and health promotion activities as well as health surveillance procedures is essential to improve health of workers and promote positive social climate at the work place.

## Introduction

Health promotion at the workplace and for workers is important. It includes promoting healthy lifestyles, the maintenance and promotion of workers’ health and working capacity, improvement of working environments and work practices [1, 2]. This is to ensure they are conducive to safety and health, as well as the development of work organization and working cultures in a direction that supports health and safety at work, promotes a positive social climate, which may enhance the productivity of the enterprise [1–3].

Companies governed by a collective bargaining agreement have a responsibility for staff to undergo routine medical screening. These medical screening done as part of health surveillance in the workplace aims to identify health risks and improve worker productivity [3]. Health surveillance procedures are usually established in the workplace to contribute to the prevention of work related ill health and to promote general health, i.e. the physical, mental and social well-being of employees [1, 3]. Health surveillance at the workplace may be established to identify work-related effects or non-work related effects of risks that workers are exposed to. Pre-employment, fitness-for-work, occupational risk, and periodic medical examinations are all well recognized health surveillance procedures [2, 3].

Workers are expose to numerous risk such as air pollution and contamination from workplace activities, handling and dismantling of e-waste, ingestion and inhalation of poisonous chemicals, gases, heavy metals or semivolatile organic compounds [4–6]. In recent years there has been a steady growth of work related psychosocial stressor characterized by strenuous work arrangements and increased job instability, which holds particularly true for the media industry [7]. These hazardous exposures and stressors have implications on the health and wellbeing of workers; periodic medical examinations and work place assessments are imperative.

However clear objectives for carrying out these medical examinations, the basis for any sound workplace programme are often absent [8]. Organizational health should be viewed as one link in the chain of organizational effectiveness. A company can be healthy for itself by growing and being efficient, healthy for its employees by offering a healthy work environment and meeting their growth needs. In addition, the company may be healthy for its customers by offering good products and services, and healthy for the community by exhibiting concern not only for its viability but also for the environment [9].

One way of making these workplace screening programmes meaningful, is to organize a health risk assessment (HRA) for staff at the workplace [10]. This is a systematic approach to collecting information from individuals that identifies risk factors, provides individualized feedback, and links the person with intervention to promote health, sustain function and/or prevent disease [10]. A typical HRA instrument obtains information on demographic characteristics (e.g. sex, age), lifestyle (e.g. smoking, exercise, alcohol consumption, diet), personal medical history, and family medical history. In some cases, physiological data (e.g., height, weight, blood pressure, cholesterol levels) are also obtained [1, 10, 11].

There have been suggestions for employers to contain health expenditures of their workers through demand management programs at the workplace [12]. These programs are designed to reduce utilization by focusing on disease prevention, work site health promotion, wellness programs, and access management. Work site health promotion is a comprehensive approach to improving health through awareness creation, health education, behavioral change, and organizational health initiatives [12].

It is critical to explain the benefits of the health risk assessment to workers, usually the health professional asks asymptomatic individuals to participate in some diagnostic or investigative procedure which may or may not seem directly beneficial to him or her. Lifestyle health risk assessments aim to identify those lifestyles which contribute to ill health and which can be modified to result in the prevention of illness, promote the health of the individual worker and garner economic benefit to the company and country [1, 2, 13]. These workplace assessments may not be done regularly by employers especially in low income setting where national incomes are low and capacity to increase labour cost are limited [14, 15].

The goal of this analysis was to provide an example of health risk assessment conducted in a large media organization in Ghana for its workers and to identify correlates of health risks among different categories of workers. A large media organization was selected to provide a baseline assessment to promote this healthful activity among employers and corporate enterprises in Ghana and other low income settings.

## Methods

This was a cross sectional study of the health risk of staff in a large corporate media organization in Accra, Ghana, conducted in May-June 2012.

### Site of study

This large corporate media organization has over 400 employees. The staff are categorized into three, Management staff, Senior staff and Junior staff. The company also has temporary staff who are outsourced from employment agencies. The institution has an offsite clinic with one permanent Senior Medical Officer (an occupational health practitioner), two Nursing Officers, two pharmacy staff and three support staff. The clinic renders out-patient curative services and attends to between 30 and 40 clients daily from 8:00 am to 5:00 pm. Medical emergencies and severe disease conditions are referred to other hospitals in Accra. Periodic health assessments are conducted by members of the health team for all categories of staff in the organization. This analysis is one such assessment conducted in 2012.

### Study population

The subjects for this analysis were categorized into three, management staff, senior staff and junior staff. Management staff include the Managing director, directors of various departments and managers of various units who are mainly engaged in administrative and supervisory duties. Senior staff include technical and administrative staff who are mainly involved in technical services associated with the electronic and print media. Junior staff are mainly support staff to the technical officers, messengers and hospitality staff who engage in housekeeping, carting, transportation and sale of media products.

### Sampling methods

All members of staff who provided consent and were available were screened and records were included in the analysis. Members of all the three categories of staff, management staff, senior staff and junior staff were involved.

### Data collection

An Abstraction form was used to collect data on the personal characteristics of staff, such as; age and sex of staff and description of staff category (i.e. Junior staff, Senior staff, Management staff) and the departments of each staff in the organization. Data on self-reported health risk (tobacco use); self-reported history of chronic disease (hypertension) and self-rated health status (as good or poor) were collected. Self reported data was compared to the clinical records for accuracy.

#### Measurements

Data were collected on weight in kg, height in cm, Body Mass Index (BMI) in Kg/m^2^, fasting blood sugar (FBS) in mmol/L, total cholesterol and HDL cholesterol in mmol/L and blood pressure in mmHg. In this analysis, normal ranges for measured health indices were: Fasting Blood Sugar 4.1–5.9 mmol/L; Total Cholesterol <5.2 mmol/L; HDL Cholesterol <1.0 mmol/L as Low, >1.55 mmol/L as High. BMI ranges, <18.5 as underweight, 18.5–24.9 as normal weight, 25–29.9 as overweight and ≥ 30 as obesity.

### Data handling

All data were treated with a high level of confidentiality. Unique identifiers and codes were employed to de-personify the participants and were used for computer-based data entry. In all cases, abstraction forms and documentations were kept securely locked. Computerized records of the survey were kept in locked files. These documents were accessible to the occupational physician only.

### Data analysis

Data from the abstraction form were entered into Statistical Package for the Social Sciences (SPSS) version 21, and analyzed. Age and sex characteristics of staff, history of chronic disease and use of tobacco were analyzed by simple frequencies, proportions, ratios and presented as tables. Measurements of health indices were analyzed by mean ± standard deviation. Outcome measures were disaggregated by staff category (MS, SS and JS). The current analysis did not assess health risk by sex of the corporate worker. Significant association between categorical outcome measures were determined with chi-square test at the 95 % confidence level.

### Ethical issues

Clearance was obtained from Management and Occupational Health Unit of the corporate media organization. No reference has been made to identify or link the organization to this analysis.

## Results

In all, there were 161 workers involved in the health risk assessment, constituting 40.3 % of the entire staff population of the organization. There were more males (112) than females (49), giving a male: female sex ratio of 2.3: 1. As shown in Table 1, the overall mean and standard deviation of the age of staff was 45.1 ± 9.6 years, it was 46.4 ± 8.8 years among males, and 42.2 ± 10.7 years in females. Senior staff were in the majority, 90 (55.9 %), and management staff were the least, 34 (21.1 %).

**Table 1:** Age and sex characteristics of staff and categories of staff involved in health assessment of the organization, Accra, Ghana

Age group	Sex of staff		Total
	Male	Female	
21- 30 years	7 (6.3)	11 (22.4)	18 (11.2)
31-40 years	22 (19.6)	11 (22.4)	33 (20.5)
41-50 years	40 (35.7)	12 (24.5)	52 (32.3)
51-60 years	43 (38.4)	15 (30.6)	58 (36.0)
Total	112 (100)	49 (100)	161 (100)
Number and staff categories involved in health risk assessment			
Staff categories	Frequency	Percent	Cumulative percent
Junior staff	37	23	23
Senior staff	90	55.9	78.9
Management staff	34	21.1	100.0
Total	161	100.0	

Among all staff, 66 (41 %) reported presence of a chronic disease condition, this report was significantly higher in senior staff (42.2 %) compared to management staff and junior staff (*p*-value = 0.001) as shown in Table 2. In all, only 10 of the 161 staff members reported ever using tobacco (i.e. Self-reported prevalence of tobacco use among staff was 6.2 %). More than a third of all senior staff (35.6 %) and management staff (35.3 %) self-reported the presence of hypertension. Close to a quarter 40 (24.8 %) of all staff self-rated their current health status as poor.

**Table 2:** Self-reported medical history and self-rated health status of staff in the organization, Accra, Ghana

Staff category	Personal medical history			
	Yes (%)	No (%)	Total (%)	*P*-value
Self-reported Chronic disease				
Junior staff	14 (37.8)	23 (62.2)	37 (100)	0.001
Senior staff	38 (42.2)	52 (57.8)	90 (100)	
Management staff	14 (41.2)	20 (58.8)	34 (100)	
Total	66 (41.0)	95 (59.0)	161 (100)	
Self-reported tobacco use				
Junior staff	5 (13.5)	32 (86.5)	37 (100)	
Senior staff	4 (4.4)	86 (95.6)	90 (100)	
Management staff	1 (3.0)	33 (97.0)	34 (100)	
Total	10 (6.2)	151 (93.8)	161 (100)	
Self-reported hypertension				
Junior staff	7 (18.9)	30 (81.1)	37 (100)	0.230
Senior staff	32 (35.6)	58 (64.4)	90 (100)	
Management staff	12 (35.3)	22 (64.7)	34 (100)	
Total	51 (31.7)	110 (68.3)	161 (100)	
Self-rated current health status				
	Good (%)	Poor (%)	Total (%)	*P*-value
Junior staff	28 (75.7)	9 (24.3)	37 (100)	0.592
Senior staff	66 (73.3)	24 (26.7)	90 (100)	
Management staff	27 (79.4)	7 (20.6)	34 (100)	
Total	121 (75.2)	40 (24.8)	161 (100)	

Overall mean ± standard deviation (SD) of BMI of staff was 27.0 ± 5.1, and was highest among management members (28.3 ± 6.6), as shown in Table 3. Overall fasting blood sugar was 4.9 ± 1.6 mmol/L and was within normal for all categories of staff. Mean total cholesterol level of all three staff categories indicated a value above the normal range expected for the age of staff members. Interestingly, the mean total cholesterol level was 5.2 mmol/L for each staff category. Mean systolic blood pressure (SBP) and mean diastolic blood pressure (DBP) were 127.8 ± 16.4 mmHg and 86.3 ± 11.6 mmHg respectively.

**Table 3:** Measured Health indices of staff per staff category in the Corporate Organization, Accra, Ghana

Staff category		Measured Health indices							
		Weight in kg	Height in cm	Body mass index of staff	Fasting blood sugar mmol/L	Total cholesterol mmol/L	HDL cholesterol mmol/L	Systolic blood pressure mmHg	Diastolic blood pressure mmHg
Junior staff *N* = 37	Mean ± SD	71.3 ± 12.4	169.6 ± 8.6	24.7 ± 3.7	4.6 ± 0.8	5.2 ± 1.3	1.2 ± 0.2	126.6 ± 13.5	88.6 ± 10.4
Senior staff *N* = 90	Mean ± SD	76.5 ± 14.5	166.8 ± 7.0	27.5 ± 4.9	5.0 ± 1.9	5.2 ± 0.8	1.2 ± 0.2	128.1 ± 17.2	85.3 ± 12.3
Management staff *N* = 34	Mean ± SD	82.2 ± 19.6	170.2 ± 5.4	28.3 ± 6.6	4.8 ± 1.1	5.2 ± 1.0	1.3 ± 0.6	128.7 ± 18.2	86.8 ± 10.3
Total *N* = 161	Mean ± SD	76.4 ± 15.7	168.2 ± 7.3	27.0 ± 5.1	4.9 ± 1.6	5.2 ± 1.0	1.2 ± 0.3	127.8 ± 16.4	86.3 ± 11.6

Normal range for Fasting Blood Sugar 4.1-5.9 mmol/L; Total Cholesterol < 5.2 mmol/L; HDL Cholesterol <1.0 mmol/L Low, >1.55 mmol/L High. BMI ranges: < 18.5 is underweight, 18.5-24.9 is normal weight, 25–29.9 is overweight and ≥ 30 is obesity

Further assessment of health risk factors among staff in Table 4 shows that, more than half of all staff (63.8 %) were obese, and almost half (49.1 %) had above normal total cholesterol levels. Diastolic blood pressure (≥90 mmHg) was above normal for 60.2 % of all staff and 20 (12.2 %) of all staff had blood glucose indicative of diabetes.

**Table 4:** High risk groups among each staff category in the Corporate Organization, Accra, Ghana

Staff category	Overweight/obesity BMI > 29.9	Fasting blood sugar > 5.9 mmol/L	Total cholesterol ≥ 5.2 mmol/L	Systolic blood pressure ≥ 140 mmHg	Diastolic blood pressure ≥ 90 mmHg
	*n* (%)	*n* (%)	*n* (%)	*n* (%)	*n* (%)
Junior staff *N* = 37	16 (43.2)	3 (8.1)	17 (45.9)	6 (16.2)	25 (67.6)
Senior staff *N* = 90	63 (70)	13 (14.4)	47 (52.2)	29 (32.2)	50 (55.6)
Management staff *N* = 34	24 (70.6)	4 (11.8)	15 (44.1)	10 (29.4)	22 (64.7)
Total *N* = 161	103 (63.8)	20 (12.4)	79 (49.1)	45 (28)	97 (60.2)

Over 70 % of senior staff and management members were either overweight or obese, and close to a third had systolic blood pressure ≥ 140 mmHg. More than half of senior staff had above normal total cholesterol levels and above normal diastolic blood pressure.

## Discussion

This health risk assessment of staff in a large corporate media organization in Accra, Ghana provides an example of the conduct of a healthful corporate activity by employers to ensure health and safety at the work place and enhance productivity [1–3]. In all 40 % of total staff population were involved in the assessment. It was voluntary, some workers refused consent to participate and others were unwilling for the blood samples to be taken, while others were absent on official assignments (i.e. 239; 60 %). The sex characteristics of the workers indicate more males than females with a male: female sex ratio of 2.3: 1 which is not surprising as men dominate in most corporate organizations and institutions in Ghana [16]. The current analysis however, did not assess health risk by sex of the corporate worker; and does not speak to sex differences in occupational risk of the corporate worker; this was not an objective of the study.

It is essential that employers especially in low income settings identify health risk assessment in the workplace as a cost saving measure to improve productivity. It may increase labour cost but the benefits garnered from productive workforce may be greater [13]. Early detection of risk provides opportunity to implement measures to prevent development of health complication and loss of productivity [1, 6, 7]. In this review close to half of the workers self-reported history of a chronic disease and almost a quarter self-rated their overall state of health as poor. Demand management programmes at the workplace designed to focus on disease prevention and health promotion including work site health promotion, wellness programmes, and improved access to health care services are important. Comprehensive health-promoting activities such as creating awareness, health education, behavioral change communication, and organizational health initiatives to engender increased physical activity is imperative [7, 12].

One critical observation from this assessment was that 66 (41 %) of staff self-reported history of chronic disease and 51 (32 %) self-reported hypertension, however measured blood pressure indicated the overall prevalence of diastolic blood pressure to be 60 % . The health implication is that more staff are unaware of being hypertensive. This finding conforms to that of the World Health Organization’s Study on Global AGEing and Adult Health (SAGE Wave 1), nationwide survey among older person 50 years and above in Ghana, which showed that the prevalence of self-reported hypertension was 14 %, while the prevalence of measured systolic and/or diastolic hypertension was 51 %. It indicated further that among the hypertensive, the percentage of persons whose treatment is effective was very low (4 %), and that most older adults who are hypertensive are not on treatment (83 %) [17]. The major point being made by this comparison of the results is that health risks are not always identified by individuals and that self-reported ill-health tends to underestimate the prevalence of measured disease [18, 19].

Apart from high blood pressure, other health risks were identified in the workers. More than half of all staff (64 %) were obese, and almost half (49 %) had above normal total cholesterol levels and 12 % had blood glucose indicative of diabetes. This indicates the burden of risk in this corporate worker group which otherwise would have gone undetected. In this relatively younger and working population, health risk assessment in the work place provides a great opportunity to identify health risk and link persons at risk to health care. The adoption of regular educational sessions with appropriately selected health topics addressed by experts, departmental health education sessions, health and safety meetings, training of first aid personnel and peer educators are measures that could improve health of workers and promote positive social climate at the work place [1, 10]. In addition, aerobic sessions at the workplace, biannual corporate games consisting of interdepartmental games and inter-organization games may be useful measures to promote health at the workplace.

Health risks varied across the different categories of health workers. Senior staff and management members had relatively higher prevalence of being overweight or obese, high systolic and/or diastolic blood pressure, above normal total cholesterol levels and above normal fasting blood sugar. This observation may be associated with the work practices and habits of these senior level staff whose duties are more sedentary and tend to be physically inactive. They may be more exposed to attending meetings and conferences, these make it more difficult for one to maintain the usual dietary habits and practices. Health surveillance at the workplace is essential to identify work-related effects or non-work related effects of risks that workers are exposed to. Pre-employment, fitness-for-work, occupational risk, and periodic medical examinations are all well recognized health surveillance procedures [1–3].

For the employer in low income setting, the margin to increase labour cost is limited and poses a real challenge [14, 15] and any activity- no matter how useful- may be looked at primarily from the cost it imposes on the organization. In settings where enforcement of law on corporate responsibility are not stringent, some employers may ignore work place health assessment and other recognized health surveillance procedures.

### Limitations

The under- or over-estimation of chronic disease/risk through self-report is of great concern for epidemiological studies and surveys [18]. This health risk assessment relied on individual submissions and the self-report of health conditions, (such as hypertension), is likely to result in underestimation of prevalence rates compared to measured rates [19]. In addition, this paper presented only cross sectional observations. We believe the paper would have been much stronger if an intervention expected to improve the quality of the workers’ health had been instituted and assessed to determine the effect. The analysis however, provides useful baseline information on health risks among workers for corporate organizations in Ghana to promote health among workers and the work place.

## Conclusion

The assessment reveals that more staff have high health risks they are not aware of and that health risks varied across the different categories of health workers. Health risks are higher among senior staff and management members. Adoption of regular health educational and health promotion activities as well as health surveillance procedures to identify risk among all category of workers are essential to improve health of workers and promote positive social climate at the work place.
